# Subjective Age and Financial Exploitation Vulnerability in Older Adults: A Cross-Lagged Analysis

**DOI:** 10.1093/geroni/igaf122.105

**Published:** 2025-12-31

**Authors:** Yoav Bergman, Gali Weissberger

**Affiliations:** Ashkelon Academic College, Ashkelon, HaDarom, Israel; Bar-Ilan University, Ramat Gan, HaMerkaz, Israel

## Abstract

During the past decade, research has demonstrated the importance of positive views of the aging process and of one’s own aging self for older adults’ physical, psychological, and social well-being. Recently, studies have demonstrated that older adults who feel younger than their chronological age (i.e., a young subjective age) reported reduced vulnerability for financial exploitation (FEV), as well as with additional related factors such as financial self-efficacy. However, such studies were cross-sectional, and little is known regarding the causal relationship between subjective age and FEV. The current study employs a cross-lagged model which examines both constructs over a period of six months. Data was collected from 82 older adults (Mage= 68.82, SD = 5.44, range 60-86) who filled out scales assessing FEV and subjective age at baseline (T1) and six months later (T2). Results indicated that FEV at T1 predicted an older subjective age at T2, whereas the association between subjective age at T1 and FEV at T2 was not significant. The discussion highlights the important connection between personal subjective views of aging and the risk for financial exploitation in older populations and suggests theoretical and practical implications of this connection.

